# Perspectives for Applying G-Quadruplex Structures in Neurobiology and Neuropharmacology

**DOI:** 10.3390/ijms20122884

**Published:** 2019-06-13

**Authors:** Sefan Asamitsu, Masayuki Takeuchi, Susumu Ikenoshita, Yoshiki Imai, Hirohito Kashiwagi, Norifumi Shioda

**Affiliations:** 1 Department of Genomic Neurology, Institute of Molecular Embryology and Genetics, Kumamoto University, 2-2-1 Honjo, Chuo-ku, Kumamoto 860-0811, Japan; s-asamitsu@kumamoto-u.ac.jp (S.A.); masayuki.takeuchi@univcorp.co.jp (M.T.); ikenoshita@kumamoto-u.ac.jp (S.I.); 192y2013@st.kumamoto-u.ac.jp (Y.I.); 2 Universal Corporation Co., Ltd., 2-13-6 Noritake-Higashi, Gifu 502-0929, Japan; kswgh@univcorp.co.jp; 3 Department of Neurology, Graduate School of Medical Sciences, Kumamoto University, 1-1-1 Honjo, Chuo-ku, Kumamoto 860-8556, Japan

**Keywords:** G-quadruplex, DNA, RNA, neurobiology, neurological disease

## Abstract

The most common form of DNA is a right-handed helix or the B-form DNA. DNA can also adopt a variety of alternative conformations, non-B-form DNA secondary structures, including the DNA G-quadruplex (DNA-G4). Furthermore, besides stem-loops that yield A-form double-stranded RNA, non-canonical RNA G-quadruplex (RNA-G4) secondary structures are also observed. Recent bioinformatics analysis of the whole-genome and transcriptome obtained using G-quadruplex–specific antibodies and ligands, revealed genomic positions of G-quadruplexes. In addition, accumulating evidence pointed to the existence of these structures under physiologically- and pathologically-relevant conditions, with functional roles in vivo. In this review, we focused on DNA-G4 and RNA-G4, which may have important roles in neuronal function, and reveal mechanisms underlying neurological disorders related to synaptic dysfunction. In addition, we mention the potential of G-quadruplexes as therapeutic targets for neurological diseases.

## 1. Introduction

The most studied form of DNA is the right-handed helix, termed the B-form. In 1953, based on X-ray crystallography data, Watson and Crick proposed the B-form DNA model, a double helical structure [1]. However, it is becoming evident that non-Watson–Crick base pairing, resulting in the formation of alternative DNA secondary structures, also occurs in the genome. These non-canonical structures, termed non-B form DNA, include the left-handed helix Z-form [2], i-motif [3] and G-quadruplex [4]. The non-B form DNA structures have been shown to influence critical genetic processes such as DNA replication, transcription, recombination, and repair [5].

**Figure 1:**
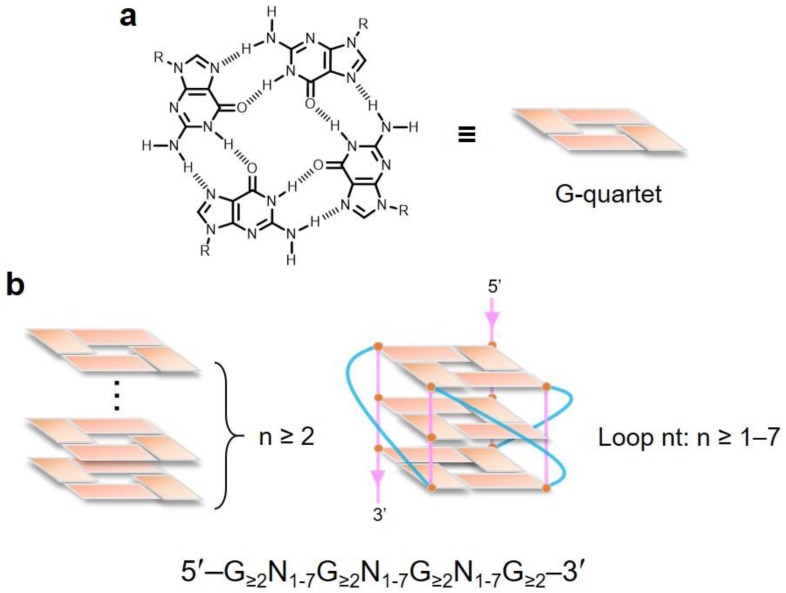
Characteristics of G-quadruplexes. (**a**) Chemical structure and schematic illustration of the G-quartet. (**b**) Schematic illustration of G-quadruplex structures. The G-quadruplex has at least two or three G-quartets, and three loops of varied length (1–7 nucleotides).

G-quadruplexes are stacked nucleic acid structures that form within G-rich DNA or RNA sequences. In 1910, it was demonstrated for the first time that a concentrated solution of guanylic acid could form a gel, suggesting that G-rich sequences may form higher-order structures [6]. Fifty years later, the characteristics of that gel have been explored in detail. X-ray diffraction analysis revealed that four guanine molecules form a square planar arrangement, so that each guanine is hydrogen-bonded to the two adjacent guanines [7], called the G-quartet (Figure 1a). Later, it was shown that single-stranded guanine-rich DNA could form a four-stranded structure in the presence of physiological salt concentrations, and that the G-quartets were stacked on top of each other to form the G-quadruplex structure. G-quadruplex can also form in the presence of four tracts with at least two adjacent guanines separated by three loop-regions with variable nucleotide composition and length within one strand. The G-rich sequence and loop size in the G-quadruplex regions vary (1–7 nucleotides) (Figure 1b) [4,8,9].

In recent years, interest in the G-quadruplex structures in vivo has increased because of their unique properties, such as high thermal stability and the presence of G-rich sequences in biologically functional regions of the genome. G-quadruplex formation may be associated with various biological functions, including DNA replication, transcription, epigenetic modification, and RNA metabolism. For example, DNA G-quadruplexes (DNA-G4) play critical roles at the telomeres, mitotic and meiotic double-strand break sites, transcription initiation sites, and replication origins [10]. In addition, RNA G-quadruplexes (RNA-G4) coordinate many steps of RNA metabolism ranging from splicing, RNA processing and transport, to mRNA translation [11]. Excellent reviews of the most up to date information regarding the roles of DNA-G4 and RNA-G4 in biological processes have been published [12,13,14,15].

In the current review, we have summarized the state of knowledge on G-quadruplexes derived from the available topological evidence and bioinformatics analysis. We have also discussed the putative roles of DNA-G4 and RNA-G4 in neurobiology, and their potential as novel therapeutic targets in neurological disorders.

## 2. Topological Analysis of G-Quadruplex

The formation of G-quadruplex is influenced by several factors, including the presence of binding cations, temperature, strand stoichiometry, orientation, and loop size [16,17]. The general trend of DNA-G4 stabilization by monovalent cations is K^+^ >Na^+^ > Li^+^; these cations are located in the center of the G-quartet [18]. Similar to DNA-G4, the central building block of RNA-G4 is the G-quartet. The stability of RNA-G4 depends on the presence of monovalent cations, mainly K^+^, e.g., in the telomeric repeat-containing RNA and neuroblastoma RAS viral oncogene homolog RNA [19]. 

**Figure 2:**
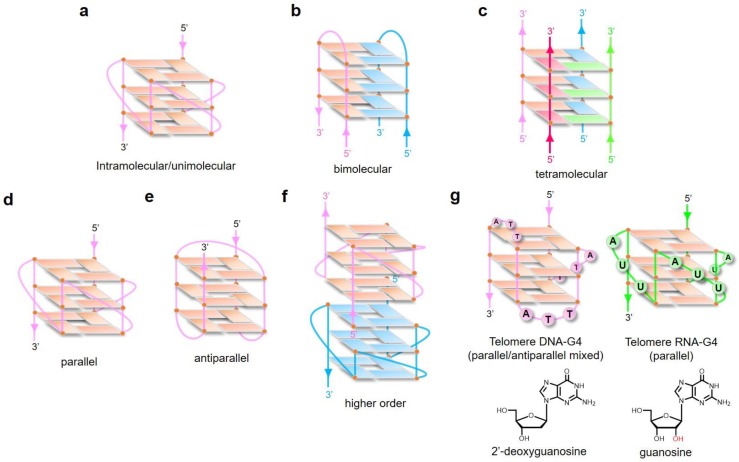
The topological variety of G-quadruplex structures. Schematic illustration of intramolecular/unimolecular (**a**), bimolecular (**b**), tetramolecular (**c**), parallel (**d**), antiparallel (**e**), and higher-order (**f**) G-quadruplexes. (**g**) Schematic illustration of telomere DNA-G4 (left) and RNA-G4 (right) in a potassium-containing solution. DNA-G4 is arranged in three G-quartet layers composed of 2′-deoxyguanosines and three TTA loops, while RNA-G4 is arranged in three G-quartet layers composed of guanosines and three UUA loops. The 2′-hydroxyl group is highlighted in red for clarity.

Numerous studies have addressed the effect of loop length and sequence on the DNA-G4 structure; adaptation of the glycosidic bond conformation (syn or anti); the number of molecules of nucleic acid involved in the structure formation, e.g., intramolecular/unimolecular (Figure 2a), bimolecular (Figure 2b), or tetramolecular structures (Figure 2c); and the relative orientation of the strands resulting in parallel (Figure 2d), antiparallel (Figure 2e), and higher-order G-quartets (Figure 2f) [20,21,22]. RNA-G4 is thermodynamically more stable and less polymorphic than DNA-G4 [23]. RNA-G4 topology is predominantly parallel and independent of the loop length, as determined by using RNA oligonucleotide libraries representing individual loop lengths of 1 to 5 nucleotides and total loop lengths of 3 to 15 nucleotides [24]. Telomere DNA-G4 and RNA-G4 structures are shown in Figure 2g. While DNA-G4 may form an equilibrium towards the Watson–Crick double-stranded conformation and is bound to histones in the nucleus, RNA-G4 may form an equilibrium towards various secondary structures (hairpins, loops, bulges, and pseudoknots) with many RNA-binding proteins. RNA may be more prone to form G-quadruplex structures in vivo than DNA, because it lacks a complementary strand. As described above, the basis of the physical topology of G-quadruplex structures has been determined by in vitro analysis. However, it should be noted that it is not yet clear how the in vitro topology translates into in vivo physiology and pathology.

## 3. Genome-Wide Analysis of G-Quadruplexes

The distribution of G-quadruplexes in the human genome is an important question that may be addressed by a genome-wide study, on the basis of (1) in silico prediction, (2) polymerase stalling analysis, and (3) antibody-mediated pull-down approach.

Many algorithms have been developed for the computational prediction of G-quadruplexes. These include Quad-Parser [25], QGRS Mapper [26], G4P Calculator [27], QuadBase [28], cGcC score [29], and G4Hunter [30]. The cGcC score may be used to predict RNA-G4 [29]. G4Hunter has been designed for DNA analysis but was also shown to be applicable for RNA analysis [30]. These algorithms may be used to predict the number of putative G-quadruplex sequences (PQS) randomly formed within a genome. The in-silico analysis has revealed that the PQS are enriched at promoters, CpG islands, 5′-untranslated regions (5′-UTRs), first exons, first exon/intron junctions, and nuclease-hypersensitive sites [25,27,30,31]. Computational predictions using Quad-Parser indicate that among 38,915 5′-UTRs of all the known *Homo sapiens* genes annotated in the National Center for Biotechnology Information database, 2922 contained one or more G-quadruplex motif [25,32]. Using the same computational prediction method, it was shown that the G-quadruplex motifs were highly correlated with the position of replication origins, 5′-UTRs, nucleosome-free regions, and CpG islands in human cells, including HeLa cells, primary fibroblasts, human embryonic stem cells, and human induced pluripotent stem cells [31]. As a limitation, these tools predict the potential formation of G-quadruplex directly from the primary DNA sequence, i.e., “G_3_ + N_1–7_G_3_ + N_1–7_G_3_ + N_1–7_G_3_ (where N = A, C, G, or T)”, with four stretches of at least three guanines separated by short stretches of other bases. Hence, the consensus sequence cannot be used to predict all DNA-G4 structures in a genome, e.g., motifs with non-guanine bulges (with a non-guanine base interrupting a three-guanine track sequence) [33]. 

Recently, a tool for the identification of potential RNA-G4s, named G4RNA screener, was reported, which was not limited by a canonical G-quadruplex motif definition [34]. G4RNA screener combined the previously established cGcC score [28], the newly described G4Hunter [29], and G4NN into a single tool. G4NN, one of the tools included in the G4RNA screener, is a novel machine learning approach trained on sequences that were investigated experimentally in previous studies. G4NN provides a score based on abstract sequence similarity, computed by a simple artificial neural network that was trained on the sequences available in the G4RNA database [35]. G4NN learns from available examples and considers both irregular and canonical G-quadruplexes. The artificial neural network takes the trinucleotide composition of a window as its input, which translates to an abstract representation of the sequence. By providing the composition of all trinucleotides, G4NN avoids bias by the classifier in considering specific trinucleotides as more important than others. 

As yet another genome-wide approach for the identification of G-quadruplex sites, polymerase stop assay was combined with next-generation sequencing [36]. The sequencing was performed in the presence or absence of G-quadruplex stabilizers, pyridostatin or K^+^. Consequently, 716,310 G-quadruplex-forming sequences stabilized by pyridostatin and 525,890 G-quadruplex-forming sequences stabilized by K^+^ were identified in primary human B lymphocytes [36]. G-quadruplex formation was also significantly associated with cancer-related genes, such as *BRCA1*, *BRCA2*, and *MAP3K8*; and oncogenes and tumor suppressors, such as *CUL7*, *FOXA1*, *TUSC2*, and *HOXB13* [34]. In another study involving the G-quadruplex stabilizer L1H1-7OTD, 9651 G-quadruplex clusters were identified by high-throughput sequencing of human genomic DNA [37]. However, these methods did not capture G-quadruplex structure formation within endogenous chromatin.

To capture G-quadruplex structure formation within endogenous chromatin, G-quadruplex chromatin immunoprecipitation-sequencing (G4 ChIP-seq) with a G-quadruplex–recognizing antibody BG4 may be used [38], e.g., to analyze the human epidermal keratinocyte HaCaT cell line [39]. In HaCaT chromatin, 10,560 G4 ChIP-seq peaks were identified with high confidence, including in cancer-related genes, such as *MYC*, *TP53*, *JUN*, *HOXA9*, *FOXA1*, and *RAC1*. Importantly, the G4 ChIP-seq peak analysis identified nucleosome-depleted regions in euchromatin, and a positive and dynamic relationship between G-quadruplex structure and transcriptional activity was noted, independent of the degree of chromatin accessibility [39]. Based on the data from genome-wide approaches for G-quadruplex identification, the number of peaks detected by G4 ChIP-seq is lower (approximately 10,000) [39] than that detected by PQS determined in silico (approximately 300,000) [25] and that identified by the polymerase stop assay (approximately 700,000) [36]. 

Two types of high-throughput transcriptome-wide analysis of RNA-G4 have been reported in studies analyzing in vitro transcripts extracted from cells: A reverse transcriptase (RT) stalling sequencing approach, termed rG4-sequencing [40], and RT stop profiling [41]. The two methods are conceptually similar (the differences in the experimental workflows have been summarized in a recent review paper [42]). Both methods were used to identify thousands of RNA-G4 structures in vitro. The rG4-sequencing approach revealed a cluster of G-quadruplex sequences that were conserved among eukaryotes [40]. The RT stop profiling was used to identify 4935 overlapping and 7852 non-overlapping RNA-G4 regions in two human cell lines, HEK293T and HeLa cells [41]. While RNA-G4 readily assembles in vitro, the structure may be unfolded in vivo. In vivo RNA-G4 genome-wide analysis involving a chemical probing sequencing approach, the dimethyl sulfate sequencing, followed by RT stop profiling in the presence of K^+^, was reported [41]. The approach was used to analyze mouse, human, and yeast cells, suggesting that most RNA-G4 regions were unfolded [41]. However, since this method was used to analyze the whole-cell ensemble RNA structural conformations within the reaction time frame, it may not have reflected the structural conformation of individual RNAs, the dynamic structural interconversions, molecule subpopulations, and heterogeneity [42]. To confirm the folding of RNA-G4 in vivo, it will be necessary to analyze further using different methods.

## 4. DNA-G4 and Neurological Diseases

Dysregulation of DNA-G4 is associated with human disorders, including neurological dysfunction, accelerated ageing, and increased risk of cancer development [43]. Although the role of DNA-G4 in neurophysiology has not yet been fully elucidated, the involvement of DNA-G4 in some neurological diseases has been demonstrated. As an example of the role in a human genetic disease, G-quadruplex structures of GGGGCC (G4C2) repeats form 800 to >4000 large hexanucleotide repeat expansions (HRE) in the *C9orf72* gene implicated in the pathogenesis of C9orf72 amyotrophic lateral sclerosis and frontotemporal dementia (C9ALS/FTD) [44,45]. After the HREs are transcribed, the resulting RNA forms nuclear foci that can be translated in all reading frames into dipeptide repeat proteins via a repeat associated non-AUG (RAN) translation [46,47]. Both repeat-derived RNA foci and RAN translation products have been proposed to drive the pathogenesis [48,49,50]. Further, G4C2 RNA can fold to form a highly stable G-quadruplex conformation [51], which may play important roles in RNA foci and/or RAN translation toxicity. Interestingly, G4C2 repeat-derived RNA drives phase separation in G-quadruplex conformation and assembles into RNA granules containing RNA-binding proteins (RBPs) in vitro and in U2OS cells, indicating that RNA-mediated perturbations in granule dynamics may underline cellular toxicity, possibly contributing to C9ALS/FTD pathogenesis [52].

Some classes of DNA-G4–binding helicases are involved in the unfolding of G-quadruplex. Mutations in many of these helicases causes human diseases associated with genomic instability, and transcriptional and epigenetic regulation. These include RecQ-like helicases (BLM and WRN), iron–sulfur (Fe–S) helicases (RTEL1, DDX11, FANCJ, and XPD), (Asp-Glu-Ala-His) DEAH-box helicases (DHX36 and DHX9), and switch/sucrose nonfermentable (SWI/SNF)-like helicase ATRX [53,54]. Among the helicases mentioned, ATRX is involved in brain function in humans. Males inheriting germline mutations in *ATRX* develop a rare, congenital neurodevelopmental condition associated with impaired intellectual ability, the X-linked alpha thalassemia intellectual disability (ATR-X) syndrome [55,56].

Considering germline mutations of *ATRX*, ATR-X syndrome is characterized by various clinical manifestations, including severe intellectual disability, facial dysmorphism, genital abnormalities, and epileptic seizures [57]. ATR-X syndrome is a very rare intellectual disability syndrome, with an incidence of approximately 1/100,000 [57]. The causative gene, *ATRX*, encodes the SWI/SNF-like helicase protein ATRX, which contains two signature motifs: (1) The ATRX–DNMT3–DNMT3L domain, which binds to histone H3 tails and the modified histone H3K9me3 [58,59,60] and (2) helicase subdomains that confer ATPase activity [61,62]. In a cell, ATRX is localized to pericentric heterochromatin via the interaction between the ATRX–DNMT3–DNMT3L domain and histone H3K9me3 [59,60]. As determined by genome-wide analysis, ATRX is detected at a wide range of tandem repeats throughout the genome, including rDNA repeats, telomeric repeats, pericentric repeats, minisatellites, and endogenous retroviral sequences [63,64]. Interestingly, ATRX is also enriched at G-rich variable-number tandem repeats, including DNA-G4, as determined by ChIP-seq analysis of primary human erythroid cells and mouse embryonic stem cells [64]. Recombinant ATRX protein binds to *Oxytricha* telomeric repeat DNA [64]. Furthermore, ATRX functions as a high-affinity RBP that directly interacts with Xist A-repeat RNA [65] and telomeric repeat-containing RNAs [66] in vitro. However, the exact domain of ATRX that binds G-quadruplex has not yet been identified.

ATRX function in the brain remains unclear. To address that issue, some researchers have analyzed brain samples of genetically modified *Atrx* mice. Some imprinted genes are up-regulated in the forebrain of an *Atrx* conditional knockout mouse [67,68], suggesting that ATRX silences the active allele by an unknown mechanism. In an *Atrx*^ΔE2^ mouse engineered to lack the *Atrx* exon 2 [69,70], the imprinted gene *Xlr3b* was significantly overexpressed. ATRX and DNA methyltransferases (DNMTs) are enriched at G-quadruplex in the *Xlr3b* CpG island, and ATRX binds to parallel G-quadruplex in the *Xlr3b* CpG island, where it regulates the *Xlr3b* expression. Conversely, reduced ATRX levels at certain sites are accompanied by reduced DNMT levels and substantial DNA demethylation, suggesting that ATRX regulates epigenetics, DNA methylation, by DNMT recruitment to G-quadruplexes [71]. Interestingly, Xlr3b protein is a component of RNA granules, which inhibit mRNA transport into the neuronal dendrite. In addition, neuron-specific *Xlr3b* transgenic mouse exhibits memory deficits, indicating that aberrant neuronal *Xlr3b* expression partially affects dendritic mRNA transport and learning behavior in the *Atrx*^ΔE2^ mouse [71]. In addition to the role of ATRX in these epigenetic modifications, especially ones of the imprinted genes in the brain, several studies on the additional roles of the protein have been reported, e.g., telomere maintenance, DNA replication, and heterochromatin silencing in mammals [72]. Further studies are required to reveal the role of ATRX interaction with regions bearing the G-quadruplex motifs in the brain.

## 5. RNA-G4 and Neurological Diseases

It has been suggested that RNA-G4 plays a critical role in the translocation and translation of neuronal mRNAs in the axon, dendrite, and dendritic spine. Neuronal mRNAs are transported to the axon and dendrite, and are locally translated at the synapse. The mRNA transport is related to synaptic plasticity, and underlies learning and memory, with deregulation of this process greatly impacting neural function [73,74,75,76]. Dendritic mRNA forms a complex with RBPs and some motor proteins along the microtubules, in the form of RNA granules, transported to the dendrite. Furthermore, local protein translation of specific mRNAs takes place at the synapse. QGRS mapper, a bioinformatics tool for G-quadruplex analysis [25], revealed that approximately 30% of the best-characterized dendritic RNAs harbor the G-quadruplex motif in the 3′-UTR [77]. Representative dendritic mRNAs, i.e., those of the postsynaptic localization protein PSD-95 and calcium calmodulin-dependent protein kinase IIα (CaMKIIα), harbor a G-quadruplex structure in the 3′-UTR, and structural RNA-G4 mutations of these mRNAs inhibit delivery to the dendrite [77]. Interestingly, in a mouse harboring CaMKIIα with a deficient 3′-UTR, CaMKIIα mRNA was not transferred to the dendrite and the animal exhibited memory-learning disorder [78]. 

Transport of mRNA in the axon and dendrite is controlled by RBPs that act in trans on RNA-G4. It has been speculated that mutations in RBPs with G-quadruplex–binding ability cause neurological diseases, with a high incidence, including amyotrophic lateral sclerosis (ALS), the fragile X syndrome (FXS), and fragile X-associated tremor/ataxia syndrome. Bioinformatics analysis of G-quadruplex–binding proteins from the G4IPDB database, which contains over 200 molecules from various organisms, has been conducted [79]. Comparison of the amino acid composition of all (77) described G-quadruplex–binding proteins of *H. sapiens* revealed that they share a 20-amino acid motif of glycine and arginine residues (RGRGR GRGGG SGGSG GRGRG). Interestingly, the motif is similar to the arginine-glycine-rich (RGG) box found in the causative RBPs of neurological disorders, such as TDP-43, FUS, and FMRP [80,81]. Below, we have focused on the well-studied protein FMRP encoded by the fragile X mental retardation gene 1 (*FMR1*), the causative gene of FXS and fragile X-associated tremor/ataxia syndrome [82]. 

Individuals with FXS exhibit an intellectual disability of variable severity and display a wide array of behavioral alterations, such as hyperactivity, attention deficit, anxiety, and epilepsy. The incidence of FXS is 1 in 5000 males [83]. Most cases of FXS are caused by a large expansion of CGG repeats in the 5′-UTR of *FMR1*, and subsequent hyper-methylation of the *FMR1* gene promoter, leading to the loss of FMRP expression [84]. In the neuron, FMRP binds to synaptic polyribosomes and is an RNA granule component [85]. FMRP is a sequence-specific mRNA-binding protein that represses translation of a subset of dendritically localized mRNAs, corresponding to approximately 4% of all mRNAs in the brain [86]. FMRP binds predominantly in the coding regions of mRNA, as determined by high-throughput sequencing of RNAs isolated by crosslinking immunoprecipitation, and stalls polyribosomes on mRNAs encoding presynaptic and postsynaptic proteins implicated in psychiatric disorders [87].

Interestingly, FMRP recognizes RNA-G4 in target mRNA [88,89]. In fact, 432 mRNAs were co-immunoprecipitated with FMRP from the mouse brain, nearly 70% of which contained a G-quadruplex sequence [90]. FMRP contains four RNA-binding motifs: Three K-homology domains and one RGG box [86,91,92,93]. The RGG box is positioned in the major groove of the G-rich RNA sequence, binding it with nanomolar affinity in vitro [94]. In addition, X-ray crystallography analysis revealed that the RGG peptide stabilized the G-tetrads and facilitated G-quadruplex formation [95]. 

The ability of FMRP to bind G-quadruplex is probably linked to FXS, leading to alterations in protein translation and RNA localization. The absence of FMRP changes the production of targeting proteins that are essential for neural development and whose encoding mRNAs harbor G-quadruplex structures. Biochemical assays confirmed that FMRP functioned as a repressor of translation of their own mRNA by binding to *Fmr1* G-quadruplex in exon 13 [89,96]. The G-quadruplex–binding ability of FMRP also represses the translation of some mRNAs, e.g., *MAP1B*, *APP*, and *PP2Ac* [97,98,99,100]. FMRP binding to *MAP1B* mRNA represses translation during synaptogenesis in neonatal brain development. Elevated MAP1B protein levels lead to abnormally increased microtubule stability in the brain of an *Fmr1* knockout mouse [97]. FMRP interaction with a G-quadruplex structure located in the 5′-UTR of *Map1B* favors ribosomal stalling [98]. In addition, FMRP binds to a G-rich element in *APP* mRNA, and represses its translation [99]. Furthermore, FMRP controls the levels of PP2A by modulating the expression of its catalytic subunit, PP2Ac. FMRP appears to be a negative regulator of *PP2Ac* mRNA translation, by binding with high affinity to the 5′-UTR of the *PP2*Ac mRNA, which harbors four G-quadruplex structures. In the absence of FMRP, increased expression of PP2Ac alters actin remodeling [100]. These observations suggested the role of FMRP as a translational repressor when it binds and stabilizes a G-quadruplex structure located in the 5′-UTR of an mRNA, by preventing ribosome scanning. Ribosome profiling of mouse adult neural stem cells revealed that FMRP mainly acts as a translational repressor [101]. It has been speculated that the reduction of gene expression correlates with the stability of the corresponding RNA-G4 [102,103], indicating that RNA-G4 in the 5′-UTR of mRNAs may be a negative modulator of gene expression at the translation stage. Further, it has been recently demonstrated, using a ribosome profiling strategy, that the folding of RNA-G4 structures hinders the translation of protein-coding sequences in HeLa cells by stimulating the translation of repressive upstream open reading frames (uORFs) [104]. Indeed, depletion of the DEAH-box helicases DHX36 and DHX9, which unwind RNA-G4s, shift translation towards RNA-G4s containing uORFs, thus disturbing the translation of selected transcripts for proto-oncogenes, transcription factors, and epigenetic regulators [104].

On the other hand, some mRNA targets of FMRP, such as *Semaphorin* 3F (*Sem3F*) and *arginine vasopressin receptor V1a* mRNAs, harbor a G-quadruplex structure in the 3′-UTRs [88,105]. In *Fmr1* knockout cells, which lack FMRP, the association of *Sem3F* and *V1a receptor* mRNAs with polyribosomes is reduced [88], suggesting that in this case, FMRP could act as a translational activator or could impact the stability of these mRNAs. 

## 6. G-Quadruplex Is a Therapeutic Target for Neurological Diseases

Small molecules binding to G-quadruplex have been identified [106,107], and these molecules have therapeutic potential. G-quadruplex–binding small molecules, such as porphyrin, function as transcriptional repressors [106,107,108]. Porphyrins, PPIX and hemin, are potential candidate drugs for treating the ATR-X syndrome [71]. Importantly, PPIX and hemin are produced from 5-aminolevulinic acid (5-ALA) in vivo. Intracellular generation of porphyrins following the administration of 5-ALA significantly rescued neuronal phenotypes seen in *Atrx*^ΔE2^ mice [71]. Although potential off-target effects remain to be investigated, a novel therapeutic strategy targeting DNA-G4 in patients with the ATR-X syndrome is conceivable. Further, 5-ALA has been clinically approved as a fluorescent reagent for glioblastoma resection in Europe, Canada, and Japan [109]. Consequently, data on 5-ALA safety and pharmacology in humans are available. 

In C9ALS/FTD, molecules targeting G4C2-repeat RNA have therapeutic potential by preventing pathogenic interactions of the expanded RNA with RBPs, and/or by interfering with RAN translation. Several research groups have reported that small-molecule compounds or antisense oligonucleotides targeting the G4C2-repeat RNA are effective [110,111,112]. DNA and RNA C9orf72 HREs exist in equilibrium between two folded states, as a hairpin and G-quadruplex structure [51,110]. The small molecule 1a binds the hairpin structure of G4C2-repeat RNA and inhibits C9ALS/FTD-associated defects, RAN translation, and foci formation in induced neurons [110]. Antisense oligonucleotides targeting *C9ORF72* mRNA improve ALS-associated defects in some mouse models [111,112]. Collectively, these observations serve as a proof of principle for the development of drugs that target RNA-G4 in C9ALS/FTD.

## 7. Concluding Remarks and Future Perspectives

**Figure 3:**
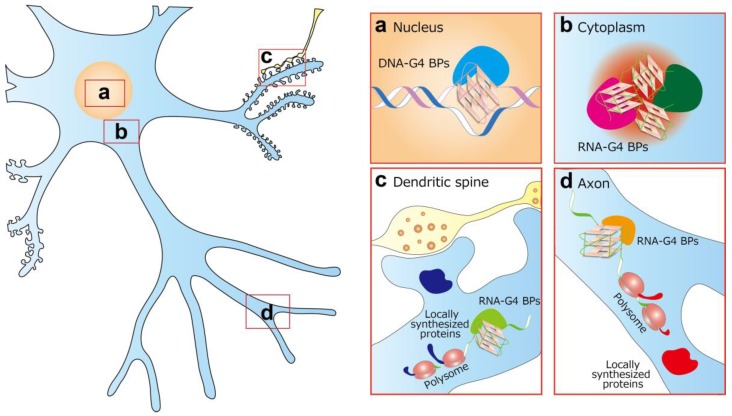
Multiple roles and pharmacological targets of G-quadruplex in the neuron. (**a**) In the nucleus, DNA-G4 may be involved in transcriptional modifications, telomere maintenance, and epigenetic modifications, together with DNA-G4 binding proteins (BPs). (**b**) In the cytoplasm, RNA-G4 is involved in the metabolism of RNA granules, including interactions with RNA-G4 BPs and phase separations. (**c**,**d**) RNA-G4 is also involved in the transport, stability, translation, and subcellular localization of dendritic (**c**) and axonal (**d**) mRNAs. A detailed understanding of these processes will inform a new therapeutic strategy for neurological diseases.

Genome-wide approaches for detecting DNA-G4 and RNA-G4 revealed the various regulatory functions of G-quadruplex structures, but the dynamic control of the folded and unfolded states data, especially in RNA-G4 analysis, require careful interpretation. For DNA-G4, some questions remain: (1) Is DNA-G4 formation different at different cellular developmental stages? (2) How and does DNA-G4 formation link to epigenetic markers? (3) How and does DNA-G4 formation regulate G4-binding proteins? In addition, it is necessary to elucidate whether DNA-G4 is involved in transcriptional modifications, telomere maintenance, and epigenetic modifications in the neuron (Figure 3a). Understanding the G-quadruplex structure and function is required for the advancement of drug development. G-quadruplex stabilizers can block the interactions between pathogenic RNA and RBPs, block abnormal phase separations, and inhibit RAN translation (Figure 3b). The effect of G-quadruplex stabilizers should also be evaluated by studying the metabolism of dendritic (Figure 3c) and axonal (Figure 3d) mRNAs, including their stability, translation, and subcellular localization. If treatments with such stabilizers could restore the normal RNA metabolism, they could represent a new avenue for the therapeutic approach to neurology in the future. Alternatively, pharmacological approaches can be used to facilitate the understanding of some roles of G-quadruplex structures in the neuron. The current knowledge represents only the tip of an iceberg, with many exciting discoveries still waiting in the “G-quadruplex World”.

## Abbreviations

DNA-G4	DNA G-quadruplex
RNA-G4	RNA G-quadruplex
PQS	putative G-quadruplex sequences
UTR	untranslated region
ChIP-seq	chromatin immunoprecipitation-sequencing
RT	reverse transcriptase
HRE	hexanucleotide repeat expansion
RBP	RNA-binding protein
C9ALS/FTD	C9orf72 amyotrophic lateral sclerosis and frontotemporal dementia
RAN	repeat associated non-AUG
ATR-X	X-linked alpha thalassemia intellectual disability
DNMT	DNA methyltransferase
CaMKIIα	calcium calmodulin-dependent protein kinase IIα
FXS	fragile X syndrome
5-ALA	5-aminolevulinic acid
